# Hammerhead ribozymes going viral

**DOI:** 10.1186/s13059-016-1007-z

**Published:** 2016-06-23

**Authors:** Christian Hammann

**Affiliations:** Ribogenetics Biochemistry Lab, Department of Life Sciences and Chemistry, Molecular Life Sciences Research Center, Jacobs University Bremen, Campus Ring 1, DE 28759 Bremen, Germany

## Abstract

An association between hammerhead ribozymes and non-autonomous, long terminal repeat retrotransposons is uncovered in plants, shedding light on the biological function of genomically encoded ribozymes.

## Introduction

In recent years, research on small nucleolytic ribozymes—RNA motifs with catalytic RNA cleavage and ligation activity—has gained new momentum by the discovery of both large numbers of known catalytic RNA (catRNA) motifs encoded in various genomes [1] and entirely novel ribozyme types [2]. Such motifs occur in genomes from all kingdoms of life but appear to be unequally distributed amongst species, and little is known about their biological function. A recent study by Cervera and colleagues [3], published in *Genome Biology*, reports the discovery of retrozymes, which are a novel class of transposable elements in plants that contain hammerhead ribozymes (HHRz), a class of small nucleolytic ribozymes catalyzing nucleotide-exact RNA cleavage and ligation [1]. The characterization of several members of the retrozyme family has revealed features that suggest a function of the catalytic HHRz motifs in retrozyme RNA replication.

## The discovery of retrozymes

Small endonucleolytic ribozymes were originally discovered in certain viroids or viral satellite RNAs, which are circular RNA (circRNA) molecules infecting plants [4]. Since their discovery, a large number of both these and novel catalytic RNA motifs have been identified in genomes [1, 5]. In their recent study, Cervera and colleagues [3] uncover HHRz motifs, encoded in plant genomes, that are associated with conserved elements which are characteristic of small, non-autonomous retroelements in plants. These include motifs from terminal-repeat retrotransposons in miniature (TRIMs) and small long terminal repeat (LTR) retrotransposons (SMARTs); additionally, retrozymes contain the conserved primer binding sites and polypurine tracts typically found in autonomous Ty3-gypsy retrotransposons. Owing to the unique combination of *retro*transposon and ribo*zyme* features, these plant elements were dubbed “retrozymes”. The association of mobile genetic elements and catalytic RNAs appears to constitute a recurrent trend in eukaryotic genomes; a catalytic RNA motif was previously found within the *Drosophila* R2 retrotransposon [6] that disrupts 28S rRNA and penelope-like retroelements were also shown to feature hammerhead ribozymes by Cervera and de la Peña [7].

## circRNA, lncRNA, catRNA… retrozymes are all of that!

**Fig. 1:**
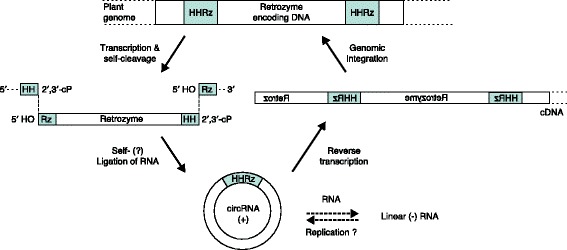
A model for the propagation of retrozymes in plants (modified from Cervera et al. [3]). Upon transcription, ligation-competent linear retrozymes are generated by hammerhead ribozyme (HHRz) self-cleavage. The covalent closure of the cleavage product yields circular RNA (circRNA) retrozymes of plus polarity (+). These serve as templates for reverse transcriptase, yielding cDNA that can be integrated into the genome by means of proteins encoded by autonomous retroelements. The observation of multimeric retrozyme RNA of minus polarity (–) suggests an additional RNA replication mechanism, involving a plant RNA-dependent RNA polymerase activity

Retrozymes are present as linear and circular RNAs in various plant tissues [3]. Following transcription of retrozyme-encoding DNA (Fig. 1), the linear RNA molecules are proposed to self-cleave one or more times, depending on retrozyme type. The catRNA activity of retrozymes with at least two HHRz motifs would lead to a ligation-competent RNA molecule. Whether circularization is auto-catalyzed in the plants by the HHRz or instead requires a protein ligase remains to be determined. In their linear form, retrozymes are long non-coding RNAs (lncRNAs).

Owing to this, no sense or antisense strands can be discerned and retrotransposon transcripts are assigned “plus” or “minus” polarities. The authors identified retrozyme transcripts of both plus and minus polarity [3], which might be explained by bi-directional transcription, as previously reported for retroelements [8]. Alternatively, Cervera et al. [3] suggest that an RNA replication event might be taking place, resulting in retrozyme transcripts of minus polarity.

Retrozymes in the form of a circRNA are readily observed in various plants and plant tissues and the circularization reaction could also be recapitulated by proteinaceous in vitro ligation [3]. The resulting circular retrozyme RNA has plus polarity (circRNA+) and features, intriguingly, a primer binding site for reverse transcription, whereas the linear form lacks the redundant R region of the LTRs required for the cDNA synthesis of conventional, linear LTR elements. The retrozyme circRNA might serve as the template for both RNA-dependent RNA polymerase (RdRP) and reverse transcriptase activities, resulting in multimeric retrozymes of minus polarity and multimeric cDNAs, respectively (Fig. 1). The latter would contain the sequences required for genomic integration by means of Ty3-gypsy retrotransposon-encoded proteins [3], thus following the conventional integration mechanism of non-autonomous retroelements. Interestingly, their integration and abundance in the genome appear to be selected against during plant domestication as retrozyme loci are drastically reduced in the genome of domesticated plants compared with that of a wild variety, at least in the case of cassava (manioc) [3].

## Do retrozyme RNAs replicate by a rolling circle mechanism?

Transposon replication by a rolling circle mechanism has been shown in the intriguing family of helitron transposons [9]. Distinct from this, the small infectious circRNAs in plants, like viroids or viral satellite RNAs, replicate by a symmetric or asymmetric rolling circle replication, hijacking the plant enzymatic machinery for RNA polymerization, cleavage (for those circRNAs without catRNA activity), and ligation, often subverting the substrate specificity of the plant enzymes in the process [4, 10].

While experimental evidence is not available yet, several observations suggest that an equivalent RNA replication step might also be operational in the case of the retrozymes [3]. For example, circular retrozymes exhibit striking structural similarities to infectious circRNAs, which lends support, albeit indirectly, to the notion of an RNA replication event in the life cycle of retrozymes. In the absence of equivalent circular DNA, the multimeric nature of retrozyme transcripts of either polarity strongly argues for a circular RNA template during transcription. Furthermore, the observation of significant sequence heterogeneity in the analyzed retrozymes suggests that they are generated by an error-prone polymerase. DNA-dependent RNA polymerase II, which acts in the rolling circle RNA replication of viroids [4], is possibly the enzyme responsible for rolling circle replication of retrozyme RNA. This would imply that its accuracy is reduced when using RNA templates. Alternatively, one of the genuine RdRPs, an enzyme class particularly prevalent in plants [8], might catalyze this reaction.

## Concluding remarks

It will be exciting to unravel in future work the identity of the key players involved in this process and further steps of the retrozyme life cycle. The importance of the HHRz motifs for these processes might be addressed using genetically traceable retrozyme variants in which catRNA activity is present or absent. It also will be of great interest to study whether the linear and circular retrozymes harbor ligation and cleavage activity, respectively, in both in vitro and in vivo systems.

## Abbreviations

catRNA, catalytic RNA; circRNA, circular RNA; HHRz, hammerhead ribozyme; lncRNA, long non-coding RNA; LTR, long terminal repeat; RdRP, RNA-dependent RNA polymerase; SMART, small long terminal repeat retrotransposon; TRIM, terminal-repeat retrotransposon in miniature.
